# Comment on Nakai, Y.; Makizako, H.; Kiyama, R.; Tomioka, K.; Taniguchi, Y.; Kubozono, T.; Takenaka, T.; Ohishi, M. Association between Chronic Pain and Physical Frailty in Community-Dwelling Older Adults. *Int. J. Environ. Res. Public Health* 2019, *16*, 1330, doi:10.3390/ijerph16081330

**DOI:** 10.3390/ijerph17010179

**Published:** 2019-12-25

**Authors:** Javad Alizargar

**Affiliations:** Research Center for Healthcare Industry Innovation, National Taipei University of Nursing and Health Sciences, Taipei 112, Taiwan; jaz.tmu@gmail.com; Tel.: +886-(02)28227101 (ext. 4215)

In their report, Nakai et al. [1] reported that chronic pain is associated with frailty in community-dwelling older adults. However, in their study, certain considerations might have been overlooked. Firstly, they used data from their previous report, but some small differences could be seen; for example, they excluded six patients with the diagnosis of dementia in the previous study [2], but in this report, they excluded seven. Reordering the exclusion criteria could help clear this out. Secondly, they mentioned that one of the exclusion criteria was “certification for long-term care”, and this needs to be further explained as different health facilities/countries have different definitions of long-term care. However, they also did not mention some conditions that might cause chronic pain, such as mild traumas and injuries. The next point to be discussed is the way they dealt with the variable “total medications used (number/day)”. This is very general and controlling for this factor, and may not cast light on the multivariate analysis. Taking painkillers might dramatically change the result interpretations.

## References

[1] Nakai Y., Makizako H., Kiyama R., Tomioka K., Taniguchi Y., Kubozono T., Takenaka T., Ohishi M. (2019). Association between Chronic Pain and Physical Frailty in Community-Dwelling Older Adults. Int. J. Environ. Res. Public Health.

[2] Makizako H., Kubozono T., Kiyama R., Takenaka T., Kuwahata S., Tabira T., Kanoya T., Horinouchi K., Shimada H., Ohishi M. (2019). Associations of social frailty with loss of muscle mass and muscle weakness among community-dwelling older adults. Geriatr. Gerontol. Int..

